# FastQAFPN-YOLOv8s-Based Method for Rapid and Lightweight Detection of Walnut Unseparated Material

**DOI:** 10.3390/jimaging10120309

**Published:** 2024-12-02

**Authors:** Junqiu Li, Jiayi Wang, Dexiao Kong, Qinghui Zhang, Zhenping Qiang

**Affiliations:** 1 College of Big Data and Intelligent Engineering, Southwest Forestry University, Kunming 650224, China; lijunqiu@swfu.edu.cn (J.L.); qzp@swfu.edu.cn (Z.Q.); 2 School of Automation Engineering, University of Electronic Science and Technology of China, Chengdu 611731, China; 202321060116@std.uestc.edu.cn (J.W.); 202411060930@std.uestc.edu.cn (D.K.); 3 Key Laboratory of State Forestry and GrassIand Administration on Forestry Ecological Big Data, Southwest Forestry University, Kunming 650224, China

**Keywords:** FastQAFPN-YOLOv8s, walnut sorting, lightweight, QAFPN, object detection

## Abstract

Walnuts possess significant nutritional and economic value. Fast and accurate sorting of shells and kernels will enhance the efficiency of automated production. Therefore, we propose a FastQAFPN-YOLOv8s object detection network to achieve rapid and precise detection of unsorted materials. The method uses lightweight Pconv (Partial Convolution) operators to build the FasterNextBlock structure, which serves as the backbone feature extractor for the Fasternet feature extraction network. The ECIoU loss function, combining EIoU (Efficient-IoU) and CIoU (Complete-IoU), speeds up the adjustment of the prediction frame and the network regression. In the Neck section of the network, the QAFPN feature fusion extraction network is proposed to replace the PAN-FPN (Path Aggregation Network—Feature Pyramid Network) in YOLOv8s with a Rep-PAN structure based on the QARepNext reparameterization framework for feature fusion extraction to strike a balance between network performance and inference speed. To validate the method, we built a three-axis mobile sorting device and created a dataset of 3000 images of walnuts after shell removal for experiments. The results show that the improved network contains 6071008 parameters, a training time of 2.49 h, a model size of 12.3 MB, an mAP (Mean Average Precision) of 94.5%, and a frame rate of 52.1 FPS. Compared with the original model, the number of parameters decreased by 45.5%, with training time reduced by 32.7%, the model size shrunk by 45.3%, and frame rate improved by 40.8%. However, some accuracy is sacrificed due to the lightweight design, resulting in a 1.2% decrease in mAP. The network reduces the model size by 59.7 MB and 23.9 MB compared to YOLOv7 and YOLOv6, respectively, and improves the frame rate by 15.67 fps and 22.55 fps, respectively. The average confidence and mAP show minimal changes compared to YOLOv7 and improved by 4.2% and 2.4% compared to YOLOv6, respectively. The FastQAFPN-YOLOv8s detection method effectively reduces model size while maintaining recognition accuracy.

## 1. Introduction

Walnuts have long been recognized as a “tree-borne oil reservoir” and a source of “plant-based protein”, valued for their nutritional and economic significance. Rich in essential fatty acids, vitamins, and minerals, walnuts are considered a beneficial component of a balanced diet. The cultivation of walnuts in China’s Yunnan Province has a history spanning over a millennium, with the region serving as an important origin and distribution center for the deeply furrowed variety. Yunnan’s diverse ecological environment provides favorable conditions for walnut cultivation, contributing significantly to its role as a major production region. However, the dense shell structure of walnuts makes it difficult to separate the shells from the kernels, making it difficult to separate walnut shells and kernels, which impacts the processing and production of walnut-related products. Therefore, implementing object detection for walnut materials can automate agricultural and food processing operations. Manual sorting relies on human screening, which is slow and costly. Automated sorting, on the other hand, uses automated equipment and a series of detection and control algorithms to automatically sort the targets, leading to faster speeds and higher efficiency. Accurate detection and localization of walnut materials can enable intelligent automation of the production line, reducing manual intervention and improving production efficiency. Currently, methods for shell–kernel separation mainly rely on magnetic separation, electrostatic separation, and physical property separation. The net selection rate of walnut kernels reaches 99.6% after the shell-breaking process [1]. Current shell–kernel separation methods can enhance the efficiency of walnut shell–kernel separation to some degree. However, due to the tight connection between the walnut shell and kernel, some kernels remain incompletely separated, affecting their utilization rate. Therefore, conducting object detection on the unsorted portions of the mixture can enhance the utilization efficiency of walnut kernels. Thus, this study aims to investigate the rapid and precise detection of unsorted shell–kernel materials using deep learning algorithms.

Deep learning is a significant area of machine learning and artificial intelligence, characterized by the use of multi-layer neural networks to process and understand complex data. By training on large-scale datasets, these models can gradually extract and learn features, enabling precise processing and understanding of various data types, including images, speech, and text. Deep learning models are now widely applied in agriculture, including maturity detection, weed detection in fields, seed classification, and nut detection [2,3,4]. Literature [5] proposes an improved YOLOv4 network with a local attention mechanism for detecting sesame weeds and explores an adaptive spatial feature fusion structure to enhance feature extraction. Literature [6] investigates an intelligent system for classifying pistachios, combining acoustic emission analysis, principal component analysis (PCA), and multilayer feedforward neural network (MFNN) classifiers. Literature [7] utilizes X-ray irradiation on pistachios to obtain relevant data and then employs a neural network for classification. The core of this approach is selecting the optimal classifier parameters using an adaptive algorithm. In summary, an increasing number of deep learning algorithms are applied in various agricultural scenarios, significantly improving productivity. Neural networks offer significant potential for applications in nut material classification. Compared to traditional classification methods, they not only enhance recognition accuracy but also simplify feature extraction.

Several methods and applications exist for detecting and classifying walnut materials. In literature [8], an improved impurity detection model based on YOLOv5 is proposed. This model adds a small object detection layer to the neck, improving the detection of smaller impurities. Additionally, the transformer encoder module replaces some convolutional blocks in the original network, enabling better capture of the image’s global information. Next, the attention module (CBAM, Convolutional Block Attention Module) is added to enhance the model’s sensitivity to channel features, facilitating the detection of prediction regions in dense objects. Finally, the GhostNet module is introduced to reduce the model’s weight. This method significantly improves recognition accuracy for small targets, but at the expense of model size and speed, with the model size increasing by 1.77 MB and detection time increasing by 4.99% compared to the original YOLOv5. Literature [9] employs region segmentation and convolutional neural networks to quickly locate and classify small walnut material targets, achieving an average recognition rate of over 94%; however, the multi-target recognition in this method relies on region segmentation and does not enable multi-target walnut material detection in a single image. Literature [10] performs walnut ripeness detection using MobileNetV3 as the backbone feature extractor of the YOLOv4 network, employing deep separable convolution instead of traditional convolution. The final improved algorithm achieves comparable accuracy and a 46.63% speedup compared to the original YOLOv4. In summary, efficient walnut material detection must balance speed, accuracy, and network efficiency to improve production efficiency.

This study proposes an object detection algorithm to identify the category of shell–kernel detachment and their spatial position when locating undetached walnuts. For embedded devices with limited computing resources and memory, reducing the size and complexity of the model ensures efficient deployment and operation. Deep learning-based object detection algorithms are mainly divided into two categories: one-stage detectors, which offer high inference speed (e.g., SSD and YOLO), and two-stage detectors, which provide high localization and recognition accuracy (e.g., Faster R-CNN, Mask R-CNN, and Cascade R-CNN). In object detection tasks, YOLO (You Only Look Once) is a highly efficient deep learning algorithm. YOLO can process images in real-time and detect multiple objects simultaneously through a single forward pass. It demonstrates satisfactory performance in detecting small and occluded targets in complex field environments and has a faster detection speed than other deep learning algorithms. However, practical agricultural scenarios require even faster and more efficient object detection networks. Therefore, there is a need to optimize and enhance YOLO for edge devices to meet the dual requirements of speed and accuracy. Literature [11] proposes a model to enhance lightweight object detection for YOLOv7 in citrus by introducing small object detection, employing the CBAM attention mechanism for the fusion and extraction of multi-scale features, and reducing the number of model parameters. Literature [12] employs a model pruning method to optimize the structure of the YOLOv4 network, resulting in improved detection accuracy for defective regions in apple images, achieving a final average classification accuracy of 92.42% and an F1 score of 94.31. Literature [13] proposes a YOLOv7 network and a data augmentation-based method for detecting oil tea fruit in complex field scenes, achieving final mAP (Mean Average Precision), Precision, Recall, F1 scores, and average detection time per image of 96.03%, 94.76%, 95.54%, 95.15%, and 0.025 s, respectively. This detection method meets the performance requirements for oil, tea, and fruit detection in natural environments. Literature [14] investigates a ShuffleNetv2-YOLOv5-Lite-E based object detection algorithm for tea leaves with one bud and two leaves and three leaves, achieving mAP values of 97.43% and 94.52% on PC and edge devices, respectively, with reductions of 1.32% and 1.75% compared to the original YOLOv5 model based on experimental results.

In summary, deep learning approaches can enhance recognition accuracy and simplify feature extraction in the agricultural field. Additionally, optimizing the network model to be lightweight can effectively enhance detection performance and speed, reduce hardware resource costs, and boost productivity. Current methods of separating walnut shells from kernels can enhance separation efficiency; however, due to the tight connection between the two, some kernels remain partially attached, negatively impacting the utilization rate. Thus, designing cost-effective, fast neural networks with reduced computational complexity holds significant potential for intelligent walnut sorting applications. This study proposes using the YOLOv8s object detection algorithm, the latest and most compact detector in the YOLO [15] family, featuring lightweight improvements and enhanced network speed.

In this study, we constructed a three-axis mobile sorting device to create a custom dataset of 3000 target images post-walnut shell breaking and proposed a FastQAFPN-YOLOv8s object detection network for fast and accurate detection of unseparated objects. The FasterNextBlock structure is built using a lightweight Pconv (Partial Convolution) operator, and the Fasternet feature extraction network is developed based on the FasterNextBlock as the backbone feature extractor for YOLOv8s. The ECIoU loss function, which combines EIoU (Efficient IoU) and CIoU (Complete IoU) structures, is utilized to expedite the adjustment of prediction frames and accelerate network regression. Finally, in the Neck network, we propose the QAFPN feature fusion extraction network to replace the PAN-FPN (Path Aggregation Network—Feature Pyramid Network) in YOLOv8s with a Rep-PAN structure based on the QARepNext (Quantization-Aware RepVGG) reparameterization framework, achieving a balance between network performance and inference speed.

## 2. Materials and Methods

During walnut processing and production, the mechanical shelling technology currently in use often fails to ensure the integrity of walnut kernels after shelling. The shelling process yields three types of materials: walnut shells, walnut kernels, and unseparated shell and kernel fragments. While initial screening can effectively separate walnut shells and kernels from unprocessed materials, identifying the unseparated type remains challenging. Therefore, it is crucial to quickly and efficiently identify the unseparated shells and kernels in the material.

In this section, we first present the walnut mixed material dataset and the experimental platform used for photographing and sorting. We then describe the image acquisition process and the lightweight object detection method used in this study.

### 2.1. Dataset Creation

**Figure 1:**
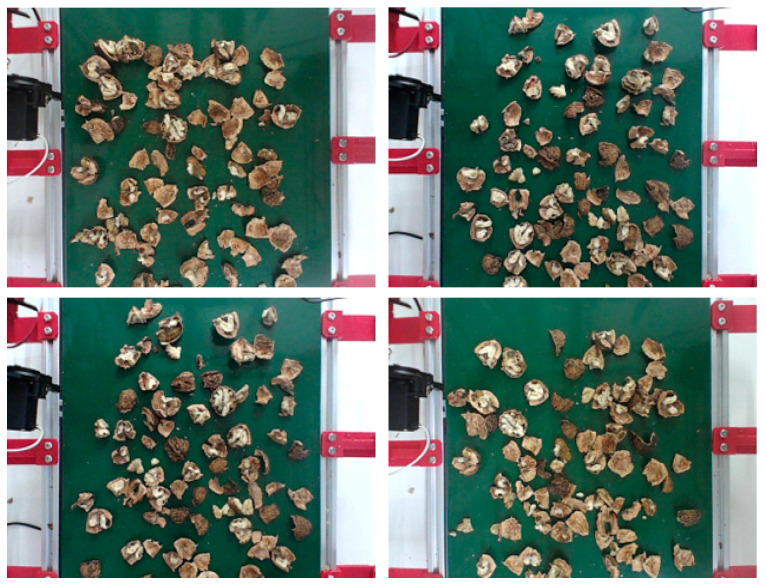
Sample images of the dataset.

Walnuts from Lincang, Yunnan Province, China, were selected for this experiment. To simulate actual processing scenarios, we randomly broke 5 kg of walnuts into pieces, including walnut shells, walnut kernels, unseparated shell and kernel fragments, and debris. The mixtures were divided into 50 groups, with samples from each group randomly placed. All sample images were captured at a resolution of 640 × 480 pixels. Sample images from part of the dataset are shown in Figure 1.

**Figure 2:**
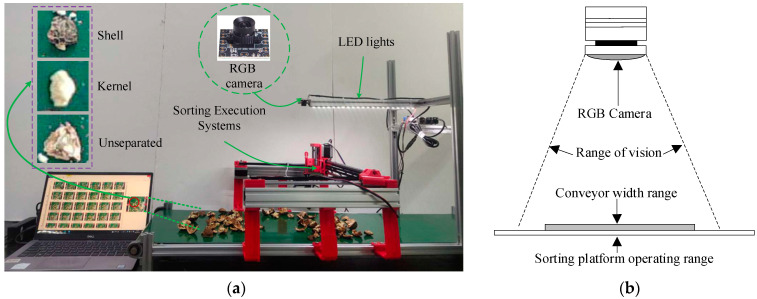
Experimental platform preparation. (**a**) Experimental platform (**b**) Field of view of the camera.

Figure 2a shows the experimental platform, which primarily consists of an optical imaging system and a sorting execution system. The system used a USB monocular camera with 90-degree distortion-free optics and a resolution of 720P to capture color images. A Raspberry Pi was used to connect the camera via USB for image capture. Figure 2b demonstrates the camera’s field of view. An LED strip was installed behind the camera to illuminate the RGB camera. The camera and LED were both mounted on an adjustable bracket. An STM32 core controller was used to automatically control the three-axis mobile sorting platform. During the experiment, the camera was mounted 34 cm above the conveyor plane. The operating range of the three-axis mobile sorting platform covered the camera’s shooting range, ensuring that all walnut material within the field of view could be sorted. The conveyor is equipped with a 42-type stepper motor and an A4988 motor driver board, which controls it to move at a speed of 0.09 m/s and stops for sorting after every 2.2 s of movement. To calculate the planar coordinates of the sorted material, a 7 × 7 tessellation grid was used for scaling, with each cell measuring 20 mm × 20 mm, corresponding to an actual pixel distance of 56 px × 56 px. The top-left corner of the image was designated as the coordinate origin, and the pixel size was converted to a distance based on the actual field of view to determine the actual location of the target.

Using the experimental platform shown in Figure 2a, we obtained datasets by capturing images. The mixed samples were divided into 50 groups, with each group randomly disrupted into 20 samples, resulting in a total of 1000 scenes. A USB monocular camera with 90-degree undistortion and a resolution of 720P was used to capture high-quality color images, controlled by the Raspberry Pi. The image sizes were all 640 × 480 pixels. The data labeling tool ‘labelimg’ was used to annotate the locations of the unseparated material fragments and to generate corresponding XML files. Using an 8:1:1 ratio, we constructed the training, validation, and testing sets, with the label type as ‘Unseparated.”

To increase the number of samples and improve the model’s generalization ability, the labeled dataset undergoes augmentations such as mirroring, rotation, contrast variation, brightness alteration, and the addition of Gaussian noise. The final dataset consists of 3000 images, divided into 2300 for training, 350 for validation, and 350 for testing.

### 2.2. Improved FastQAFPN-YOLOv8s Fast and Lightweight Detection Method

The lightweight model FastQAFPN-YOLOv8s proposed in this paper is primarily an improvement over YOLOv8s. In YOLOv8s, the backbone network consists of CSPDarkNet, and the PAN (Path Aggregation Network) architecture is utilized in the neck. Comparing the structures of YOLOv5 [13] and YOLOv8, we can see that the C3 module is replaced with the C2f module, and the convolutional structure in the upsampling phase of PAN-FPN in YOLOv5 is removed. The head network is replaced with a mainstream decoupled head that separates classification from detection, changing from an Anchor-Based approach to an Anchor-Free approach. The YOLOv8s version utilized for this improvement is a more compact and accurate model within the YOLOv8 series.

**Figure 3:**
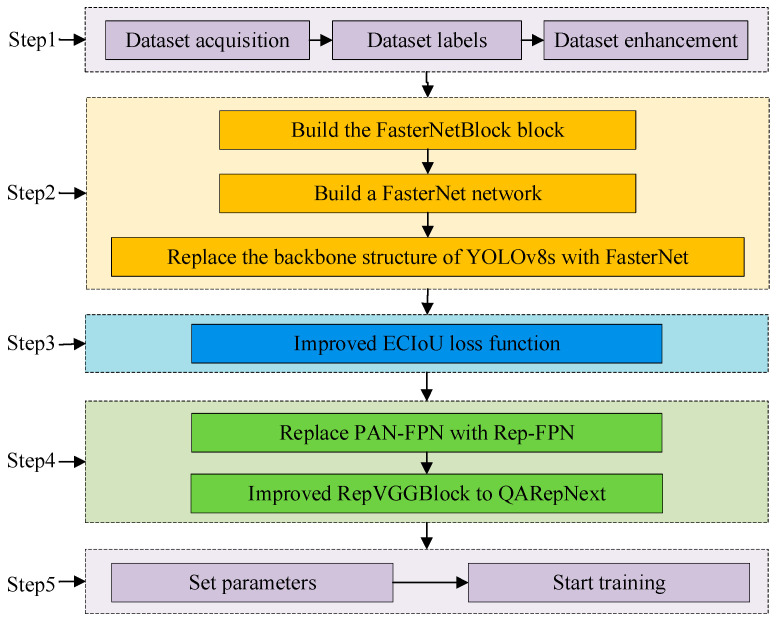
YOLOv8s improvement process diagram.

The process of improving YOLOv8s and training is shown in Figure 3.

Step 1: Obtain the dataset, label the data, and augment the dataset.

**Figure 4:**
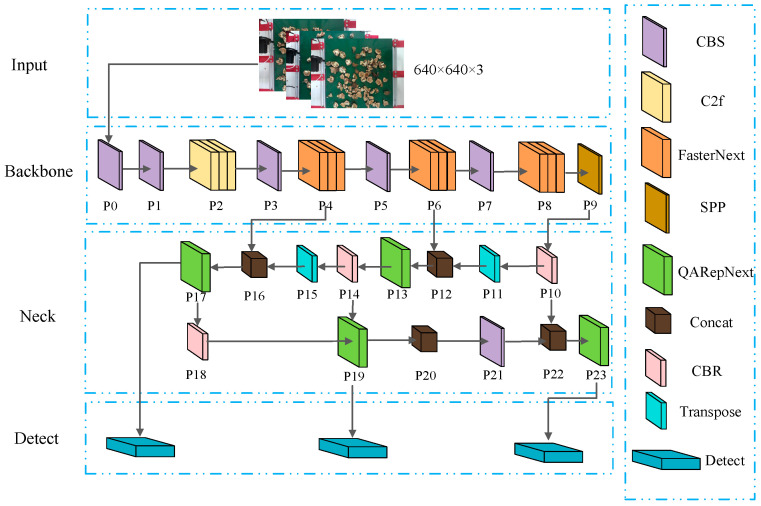
YOLOv8s specific improvement layer.

Step 2: In the feature extraction network, Fasternet is used as the backbone feature extractor. The FasterNextBlock structure was developed using the Pconv [14] operator, and the Fasternet feature extraction network was built upon it. As shown in Figure 4, P4, P6, and P8 in YOLOv8s are replaced with the lightweight FasterNext.

Step 3: In the loss function section, the ECIoU loss function, which combines EIoU and CIoU, is employed to enhance predictor frame adjustment and accelerate network regression. The process involves first using the penalty term to adjust the aspect ratio of the predictor frame to a more appropriate range, followed by minimizing the specific aspect loss to achieve a suitable value.

Step 4: In the neck network, we propose the QAFPN (QARepNext-Rep-PAN) feature fusion extraction network. We implement this by replacing the PAN-FPN in YOLOv8s with a Rep-PAN structure and utilizing the QARepNext reparameterization for feature fusion extraction. This process is illustrated in Figure 4, where CBRs are added to P10 and P14, P18 and P21 are replaced by CBRs, and the original enhanced feature extractions P13, P17, P20, and P23 are replaced with QARepNext. The reparameterized structure achieves a better balance between model performance and inference speed.

Step 5: Finally, the training parameters were configured to initiate training of the improved network.

#### 2.2.1. Building a YOLOv8s Feature Extraction Network Based on Fasternet

In computer vision, Convolutional Neural Networks (CNNs) are the prevailing architecture for fast and efficient neural network operations. Various mainstream convolutional approaches have emerged today, including swarm convolution, depth-separable convolution, and pointwise convolution. These methods are widely used in mobile-oriented and edge-oriented networks, such as MobileNets [15], GhostNets [16], ShuffleNets [17], and EfficientNet [18]. Most lightweight networks reduce model parameters and FLOPs (Floating Point Operations Per Second) by decreasing the redundancy of convolutional kernels. However, compensating for accuracy loss by increasing the network width results in more memory accesses, leading to decreased speed.

**Figure 5:**
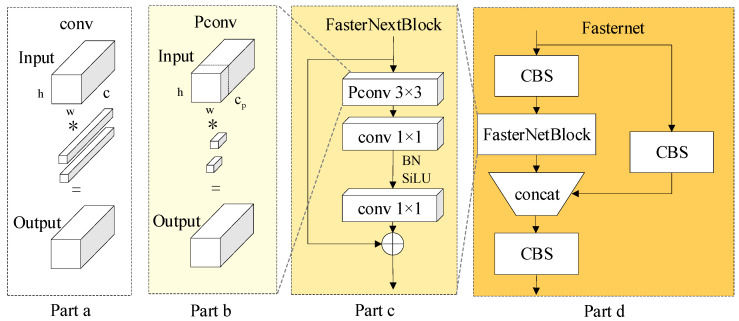
FasterNext structure construction.

In this study, the backbone network of YOLOv8s was replaced with the lightweight Fasternet series. Specifically, the three C2f backbone structures in YOLOv8s were substituted with Fasternet to enhance the network’s feature extraction capabilities, making it faster and more lightweight, as illustrated in Figure 5. Part d of Figure 5 shows Fasternet, which consists of three standard convolutional layers and multiple FasterNextBlock modules to construct a lightweight feature extraction network. The left half of the network is composed of a CBS and multiple FasterNextBlock modules, while the right half includes a CBS, with the two branches concatenated and passed through another convolutional block (CBS). In Fasternet, each FasterNextBlock module consists of one Pconv and two Conv layers, as shown in part c of Figure 5. Batch normalization (BN) and SiLU activation are applied only in the middle layers of the two Conv layers to maintain feature diversity and reduce latency. Finally, reverse residual blocks are introduced to enhance the performance of deep neural networks and prevent gradient vanishing.

In FasterNextBlock, a simple and efficient structure known as Pconv is utilized. It reduces the computational redundancy of convolution kernels while maintaining effective feature extraction with lower FLOPs and fewer memory accesses. Fasternet employs Pconv as its primary building block. Specifically, part b of Figure 5 illustrates the construction process of Pconv, which extracts features by convolving only a subset of the input channels while leaving other channels unaffected. For continuous or regular memory accesses, the first or last channel is computed as a representative of the overall feature mAP, resulting in an equal number of input and output channels.

Finally, the theoretical performance improvement of the backbone network was evaluated by calculating FLOPs and memory accesses. FLOPs refer to the number of floating-point operations and can be used to measure computational speed. A high number of memory accesses, including read and write operations, can significantly impact overall speed.

Pconv’s FLOPs:

(1) $$FLOPs\left(Pconv\right)=h\times w\times k^{2}\times {c_{p}}^{2},$$

Memory accesses:

(2) $$h\times w\times 2c_{p}+k^{2}\times {c_{p}}^{2}\approx h\times w\times 2c_{p},$$

As shown in part a of Figure 5, the FLOPs of the Conv layer are calculated in Equation (3) under the same input and output channels.

(3) $$FLOPs\left(Conv\right)=c^{2}\times k^{2}\times w\times h,$$

Memory accesses:

(4) $$h\times w\times 2c+k^{2}\times c^{2}\approx h\times w\times 2c,$$

Assuming $c_{p}=\frac{1}{4}c$, the FLOPs of the Pconv layer are only 1/16 of those of the Conv Layer, while memory accesses are 1/4 of those for the Conv Layer. Importantly, instead of reducing the original number of channels *c* to $c_{p}$, Pconv retains all channels unchanged, allowing information to flow through all channels, and using only some of the original channels $c_{p}$ for feature extraction.

As a result, enhancing the Fasternet network in the backbone can decrease network parameters, reduce the number of floating-point operations, and lower memory access. This approach facilitates a lightweight network while still maintaining efficient feature extraction.

#### 2.2.2. ECIoU-Based Bounding Box Regression Loss Function

In the original YOLOv8s object detection algorithm, the CIoU [19] (Complete Intersection over Union) bounding box loss function is utilized. This loss function incorporates three geometric factors: the overlap region, centroid distance, and aspect ratio. CIoU introduces a penalty term av to the IoU (Intersection over Union), which considers the overlap area as well as the length and width of both the predicted and ground truth bounding boxes. The formula for CIoU loss is presented in Equation (5). Here, IoU represents the intersection ratio of the predicted bounding box relative to the true bounding box, while $b,b^{gt}$ are the centroids of the predicted and true bounding boxes, respectively, used to calculate the Euclidean distance. The parameter *v* measures the consistency of the aspect ratio. As shown in Equation (6), $w^{gt}$ and $h^{gt}$ refer to the width and height of the ground truth box, while *w* and *h* represent the width and height of the predicted box. The parameter *c* is the diagonal length of the predicted bounding box and the true bounding box, and *a* is a parameter used for trade-offs, as described in Equation (7).

(5) $$\;L_{CIoU}=1-IoU+\frac{\rho^{2}\left(b,b^{gt}\right)}{c^{2}}+av,$$

(6) $$v=\frac{4}{\pi^{2}}{\left(arctan\frac{w^{gt}}{h^{gt}}-arctan\frac{w}{h}\right)}^{2},$$

(7) $$a=\frac{v}{\left(1-IoU\right)+v},$$

Although CIoU Loss considers three factors—overlap area, distance from the centroid, and aspect ratio of the bounding boxes—it cannot simultaneously increase or decrease the width and height of the predicted bounding box regression. This limitation arises because *av* does not accurately represent the true difference in width and height or their confidence. To address this issue, EIoU Loss was proposed. EIoU Loss separates the aspect ratio’s influence factor and calculates the width and height of the target frame and the anchor frame independently. It incorporates considerations for overlapping area, distance from the centroid, and the actual differences in the dimensions of the edges. The loss function for EIoU is expressed in Equation (8).

(8) $$L_{EIoU}=L_{IoU}+L_{dis}+L_{asp}=1-IoU+\frac{\rho^{2}\left(b,b^{gt}\right)}{c^{2}}+\frac{\rho^{2}\left(w,w^{gt}\right)}{c_{w}^{2}}+\frac{\rho^{2}\left(h,h^{gt}\right)}{c_{h}^{2}},$$

EIoU Loss [20] consists of three components, which include overlap loss $L_{IoU}$, center distance loss $L_{dis}$, and width-height loss $L_{asp}$. Here, IoU represents the intersection ratio of the predicted bounding box to the true bounding box, $b,b^{gt}$ denote the centroids of the predicted and true bounding boxes, and $w,w^{gt}$ represent the widths of the predicted and true bounding boxes, respectively. Similarly, $h,h^{gt}$ refer to their heights. The parameter $\rho^{2}$ is used to calculate the Euclidean distance, while *c* is the diagonal length of both the predicted and true bounding boxes. Additionally, $c_{w}$ and $c_{h}$ are the widths and heights of the smallest enclosing rectangle that can contain both bounding boxes. The ECIoU Loss, which combines CIoU and EIoU, has its loss function expressed in Equation (9).

(9) $$L_{ECIoU}=1-IoU+av+\frac{\rho^{2}\left(b,b^{gt}\right)}{c^{2}}+\frac{\rho^{2}\left(w,w^{gt}\right)}{c_{w}^{2}}+\frac{\rho^{2}\left(h,h^{gt}\right)}{c_{h}^{2}},$$

During the loss reduction process, ECIoU first adjusts the aspect ratio of the predicted bounding box to a more suitable range using the penalty term av. It then fine-tunes this ratio to the appropriate value using specific width and height loss measures, expressed as $\frac{\rho^{2}\left(w,w^{gt}\right)}{c_{w}^{2}}$ and $\frac{\rho^{2}\left(h,h^{gt}\right)}{c_{h}^{2}}$. Utilizing ECIoU can accelerate the adjustment of the predicted bounding box and enhance the regression process.

#### 2.2.3. YOLOv8s_Neck Layer Improvement Method Based on QAFPN Structure

**Figure 6:**
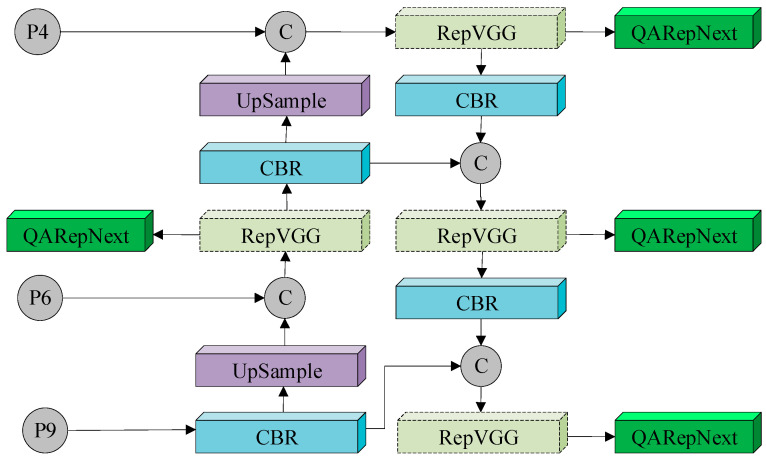
QAFPN structure construction.

To enhance the efficiency of model inference in the fusion part and achieve a better balance between accuracy and speed in the network structure, this study designed a more effective feature fusion network structure called QAFPN for YOLOv8s in the Neck layer design. Building on the principles of hardware-aware neural networks, this improved architecture is based on the Rep-PAN structure, where the RepVGG component is replaced by the QARepNext reparameterization structure. The QAFPN architecture is illustrated in Figure 6.

The Rep-PAN [21] architecture downsamples information in a top-down manner to convey strong semantic features and upsamples it in a bottom-up manner to enhance localization features. As shown in Figure 6, CBR [22] is first introduced at P10 and P14, while P18 and P21 are replaced with CBR. Enhanced feature extraction at P13, P17, P20, and P23 involves replacing RepVGG with QARepNext.

The reparameterization mechanism in the network structure provides a better trade-off between model performance and inference speed. Reparameterization allows the structure constructed during training to be equivalently transformed into another structure for inference. While the RepVGG’s reparameterized network fuses multiple branches into a single branch, it suffers from significant performance degradation during quantization. According to the literature, RepVGG [23] quantified through post-training quantization (PTQ) [24] saw a metric drop from 72.4% to 52.2%. Thus, there is a need for an effective solution to ensure better quantization performance while retaining the advantages of complex parameters.

To address this, the RepVGG module was replaced with QARepNext. Unlike the reparameterization process of RepVGG, QARepNext removes the batch normalization (BN) [25] layer from the 1 × 1 convolution and from the branches, adding an additional BN layer after the summation of the three branches to stabilize the training process. This modification resolves the quantization issues experienced with RepVGG while ensuring feature diversity and achieving lower latency. A reverse residual block was added at the end, with QARepNext replacing RepVGG at P13, P17, P20, and P23.

## 3. Result and Discussion

This study evaluated the models based on several criteria: network parameter size, model size, training duration, GFLOPs (Giga Floating-point Operations), and frame rate. The recognition accuracy of the improved object detection algorithm was assessed using mean absolute error (MAE) and average confidence. The hardware platform for this experiment, illustrated in Figure 2, consisted of an RTX 3090 GPU running on AlmaLinux with a Python 3.8 virtual environment created using Anaconda.

### 3.1. Backbone Structure Improvement Results and Comparative Analysis

The improved YOLOv8s network presented in this article uses Fasternet as the backbone feature extractor. It is trained and compared with the YOLOv8s network models that utilize MobileNet, MobileNetv2, MobileNetv3, ResNet18, ResNet50, ShuffleNetv2, and ConvNext as their backbone feature extractors. The original YOLOv8s had 11,135,987 parameters, a model size of 22.5 MB, a mAP of 95.8%, and a training time of 3.70 h. Table 1 illustrates the comparison between YOLOv8s + Fasternet and the other models. Among the various methods for improving the primary feature extractor network, YOLOv8s + ShuffleNetv2 achieved the most significant reduction in parameters and had the smallest model size. Its parameter count decreased by 42.6% compared to the original YOLOv8s, and the model size was reduced by 32%, although its accuracy dropped by 3.4%. The YOLOv8s + MobileNet model achieved the highest mAP, with an improvement of 0.9% over the original YOLOv8s. However, its parameter count increased by 3,216,640, and the model size grew by 6.5 MB. Additionally, YOLOv8s + Fasternet had the lowest training time, reducing it by 0.28 h compared to the original YOLOv8s, while decreasing the number of parameters by 1,677,968, reducing the model size by 3.1 MB, and improving the mAP by 0.3%.

Comparing the eight networks in the table, YOLOv8s + Fasternet reduces the number of parameters and model size while achieving a slight increase in mAP and a reduction in training time. Therefore, using Fasternet as a feature extractor effectively lightens the model without compromising network performance.

### 3.2. Loss Function Improvement Results and Comparative Analysis

**Figure 7:**
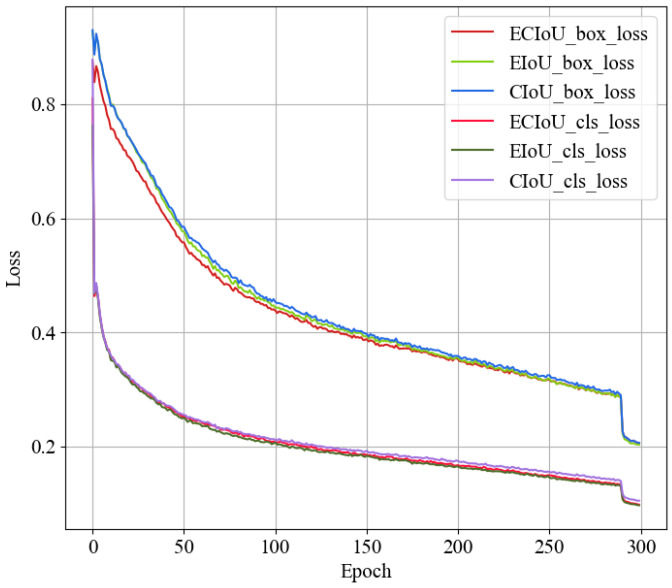
Comparison of different loss functions.

The improved YOLOv8s model presented in this paper utilizes ECIoU loss, and comparison experiments were conducted with the YOLOv8s network using CIoU and EIoU as loss functions. The comparison results are shown in Figure 7, which depicts the curves of CIoU_Loss, EIoU_Loss, and ECIoU_Loss for localization loss (box_loss) and classification loss (cls_loss) throughout the training process. The model was trained for 300 epochs, with CIoU_Loss, EIoU_Loss, and ECIoU_Loss taking 3.7 h, 4.10 h, and 3.90 h, respectively. The box_loss decreased to 0.206, 0.203, and 0.205, while the cls_loss decreased to 0.105, 0.09, and 0.098, respectively.

The ECIoU_Loss proposed in this paper is 0.49% lower in localization loss and 6.7% lower in classification loss compared to CIoU_Loss. The difference between ECIoU_Loss and EIoU_Loss is 0.002 for localization loss and 0.008 for classification loss, while requiring 0.2 h less training time. Therefore, the ECIoU loss function not only ensures effective loss reduction but also minimizes the convergence time for the predicted bounding boxes.

### 3.3. Improvement Results and Comparative Analysis of Feature Fusion Network

To validate the effectiveness and superiority of the QAFPN fusion network, this section compares the YOLOv8s backbone network, modified with improved loss functions, against several additional feature fusion networks. Model A, denoted as YOLOv8s + Fasternet + ECIoULoss, is tested with QAFPN, BiFPN, GhostSilmFPN, AsymptoticFPN, and GiraffeDetFPN feature fusion networks. The results are presented in Table 2.

The parameter count of A + QAFPN is the lowest, with only 6,071,008 parameters. Additionally, it has the smallest model size at just 12.3 MB and the shortest training time of only 2.49 h. A + BiFPN achieves the highest mAP at 95.6%, but its parameter count, model size, and training duration exceed those of A + QAFPN by 3,387,035 parameters, 6.8 MB, and 0.34 h, respectively. Notably, A + AsymptoticFPN closely matches A + QAFPN in performance, as its mAP is only 0.1% higher. However, A + AsymptoticFPN has a higher parameter count, model size, and training time compared to A + QAFPN. Therefore, the QAFPN feature fusion network represents a more lightweight option.

### 3.4. Ablation Experiments

To verify the effectiveness of the improvements, separate experiments were conducted for different enhancement modules sequentially, with modifications added incrementally. The experimental results are shown in Table 3, where A represents YOLOv8s, B is FasterNet, C is ECIoULoss, D is Rep-PAN, and E is QAFPN. GFLOPs refer to the number of floating-point operations per second in billions, while mAP represents the average accuracy rate. Speed is the result demonstrated during the training of the object detection network, calculated by summing the pre-processing, inference, loss, and post-processing times per image. To calculate the improved network detection speed, a 4 s video of walnut material with 127 frames was analyzed, and its average frame rate is presented in the rightmost column of Table 3.

a. The number of computational redundancies and memory accesses has been reduced by improving the backbone network. The experimental results are shown in Experiment 2 of Table 3. Compared to the original model, the number of model parameters decreased by 1,677,968, GFLOPs decreased by 4.1, training time decreased by 0.28 h, model size decreased by 3.4 MB, and speed decreased by 3.5. Additionally, the mAP and frame rate improved by 0.3% and 4.3 fps, respectively.

b. After improving the loss function, the convergence speed of the predicted frames has increased. The experimental results are shown in Experiment 3 in Table 3. Compared to the original model, the number of model parameters and GFLOPs remain unchanged, while the training time increases by 0.2 h. However, the mAP improves by 0.9%, Speed decreases by 3.9 ms, and the frame rate increases by 1.1 fps.

c. After enhancing the neck component, the FPN in the original YOLOv8s was upgraded to an improved Rep-PAN based on quantization-aware QARepNext to achieve a balance between performance and inference speed. The experimental results are shown in Experiment 4 of Table 3. Compared to the original model, the number of model parameters, GFLOPs, and model size were substantially reduced. The number of model parameters decreased by 2,329,888, GFLOPs decreased by 6.4, and model size decreased by 4.7 MB. The frame rate significantly improved by 18 fps, while Speed decreased by 3.9 ms and mAP increased by 0.2%. However, training time increased by 0.75 h.

d. Experiments were conducted by incrementally adding improvements one at a time. First, we trained the model by sequentially incorporating YOLOv8s, FasterNet, and ECIoULoss. The experimental results are shown in Experiment 5 of Table 3. Compared to YOLOv8s + FasterNet, the model in Experiment 5 is faster, with a 0.2 ms reduction in Speed and a 6.9 fps increase in frame rate, but it has a 0.32 h increase in training time and a 0.7% decrease in mAP. Next, YOLOv8s, FasterNet, ECIoULoss, and Rep-PAN were included for training, and the experimental results are shown in Experiment 6 of Table 3. The final results show substantial improvements in all metrics compared to Experiment 5, except for a slight decrease in accuracy. Relative to Experiment 5, the number of model parameters decreased by 3,386,243, GFLOPs decreased by 11.7, training time decreased by 1.19 h, model size decreased by 10.1 MB, Speed decreased by 0.9 ms, and frame rate improved by 3.4 fps, although there was a small decrease in mAP of 1.4%.

e. Finally, YOLOv8s, FasterNet, ECIoULoss, and QAFPN were combined to create the FastQAFPN-YOLOv8s model, which is the lightest and meets accuracy requirements. The experimental results are shown in Experiment 7 of Table 3. Compared to the model in Experiment 6, the training time and model size were reduced by 0.06 h and 0.1 MB, respectively. The mAP and frame rate improved by 0.2% and 0.5 fps, respectively.

In summary, most metrics show positive improvements with incremental training of the model. The final model demonstrates a 45.5% reduction in the number of model parameters, a 32.7% decrease in training time, and a 45.3% decrease in model size compared to the original YOLOv8s. However, there is a slight loss in accuracy, with a 1.2% drop in mAP. The improved Speed is only 1.3 ms, representing a reduction of 74.6%. The model achieved a frame rate of 52.1 FPS, a 40.8% improvement over the original YOLOv8s. Therefore, the FastQAFPN-YOLOv8s model is the most lightweight and fastest option while maintaining acceptable accuracy.

### 3.5. Model Comparison

**Figure 8:**
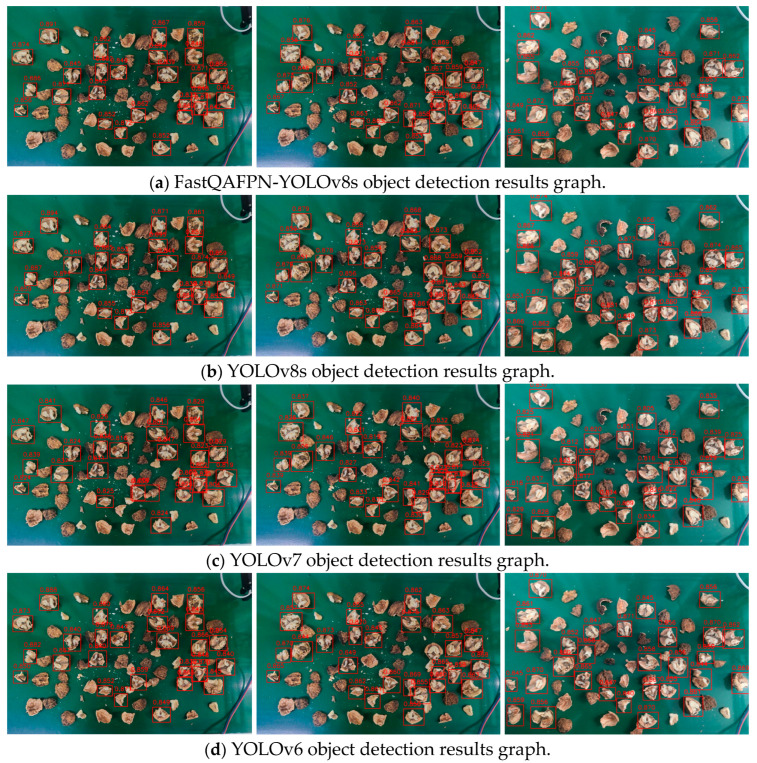
Graph of recognition results of different models.

To verify the effectiveness of the FastQAFPN-YOLOv8s model, we trained it alongside the original YOLOv8s and two earlier versions of YOLO on the same dataset. The performance of the improved network was evaluated by comparing the average confidence of detected objects and network parameters. The recognition results are displayed in Figure 8, where the red boxes and numbers indicate the detected objects and their confidence levels. Figure 8a–d shows the results for FastQAFPN-YOLOv8s, YOLOv8s, YOLOv7, and YOLOv6, respectively.

FastQAFPN-YOLOv8s and YOLOv8s exhibit similar accuracy, with a difference of less than 1%. YOLOv6 has the lowest accuracy, with an average difference of about 5% compared to FastQAFPN-YOLOv8s. The comparison results for the four models are summarized in Table 4. The size of the improved model is reduced by 10.1 MB compared to the original YOLOv8s, and it is reduced by 59.7 MB and 23.9 MB when compared to YOLOv7 and YOLOv6, respectively.

While the average confidence and mAP slightly decreased compared to the original YOLOv8, the frame rate improved by 14.6 fps. The average confidence and mAP of the improved model changed little compared to YOLOv7, but they improved by 4.2% and 2.4% compared to YOLOv6, respectively, with a significant increase in frame rate. The frame rate is 15.67 fps and 22.55 fps higher than that of YOLOv7 and YOLOv6, respectively.

In summary, FastQAFPN-YOLOv8s has the smallest model size, the highest frame rate, and higher average confidence than both YOLOv6 and YOLOv7, with only a 0.4% difference from YOLOv8s. The mAP is greater than that of YOLOv6, and the differences with YOLOv7 and YOLOv8s are just 0.3% and 0.9%, respectively. Although the accuracy of FastQAFPN-YOLOv8s is similar to that of the other versions, it stands out as the most lightweight and fastest model, making it the most suitable choice for walnut material sorting devices.

In order to verify the effectiveness of the lightweight improvement of the model in this article, the Raspberry Pi 5B embedded platform was used to deploy the YOLOv8s original model and the proposed model using Pytorch, Onnx, Ncnn, and Tfile. The frame rate data obtained from the test are shown in Table 5. The frame rate of the model proposed in this article is higher than that of the original YOLOv8s model in all four deployment modes, with the highest frame rate of 15.1 frames per second achieved in the Ncnn deployment mode.

## 4. Conclusions

Walnut fragment detection has significant potential for applications in agriculture and food processing. Efficient detection and localization of walnut fragments can enhance the automation of walnut production lines, reduce manual labor, and improve production efficiency. Additionally, this detection technology aids in the quality control of walnut products, ensuring that the final items meet the required standards.

In this paper, we propose an object detection network named FastQAFPN-YOLOv8s, designed for the rapid and accurate detection of walnut fragments that have not been separated from their shells and kernels after cracking. This method employs Fasternet as the backbone feature extractor and utilizes the lightweight Pconv operator to construct the FasterNextBlock structure. Furthermore, we use an ECIoU loss function, which combines EIoU and CIoU structures, to accelerate the tuning of prediction boxes and network regression. In the neck of the network, we propose using QAFPN as the feature fusion extraction network to balance performance and speed. Comparison experiments were conducted with different backbones, loss functions, and models, demonstrating that the FastQAFPN-YOLOv8s network model has 6,071,008 parameters, a training time of 2.49 h, a model size of 12.3 MB, and an mAP of 94.5%, with a frame rate of 52.1 FPS. Compared to the original YOLOv8s, the model parameter count was reduced by 45.5%, training time decreased by 32.7%, and model size was reduced by 45.3%. Although there was a slight decrease in accuracy (with mAP dropping by 1.2%), the improved speed was significant, with inference time reduced by 74.6% to only 1.3 ms. The frame rate of the model reached 52.1 FPS, representing a 40.8% improvement over the initial YOLOv8s.

The proposed method provides a lightweight and efficient solution for sorting unsorted walnut materials while maintaining high accuracy, thereby significantly enhancing production efficiency. However, the detection of walnut fragments still faces various challenges in practical production processes. The diverse shapes of walnut fragments increase the complexity of object detection, requiring algorithms to be sufficiently flexible to adapt to different shapes and sizes. Additionally, walnut fragments may occlude or stack on top of each other during production, making some fragments difficult to detect. Changes in lighting and environmental conditions during production may also affect image quality and, consequently, detection accuracy.

Future research can explore detection algorithms for walnut fragments of various shapes and sizes, utilizing multi-scale object detection techniques such as feature pyramid networks or multi-scale prediction methods. To address occluded and stacked walnut fragments, research can investigate deep learning-based multi-object tracking techniques combined with object detection and instance segmentation to more accurately localize and segment these fragments. For detection algorithms under varying lighting and environmental conditions, data augmentation strategies such as contrast enhancement and brightness adjustment can be introduced during model training to enhance the model’s adaptability. Additionally, adversarial training methods can be explored to improve the model’s robustness to variations in lighting and environmental conditions.Research on optimizing deep learning models and lightweight algorithms can continue to explore model compression and pruning techniques to reduce model parameters and computational complexity while maintaining high detection accuracy. Techniques such as quantization and distillation can further compress model size and increase operational speed.

In summary, the FastQAFPN-YOLOv8s model proposed and validated in this study not only provides a lightweight and efficient solution for walnut fragment detection but also lays a solid foundation for future research aimed at achieving more efficient and precise object detection in complex production environments.

## Figures and Tables

**Table 1:** Comparison of training parameters of YOLOv8s combined with different lightweight backbone features extraction networks.

Network Model	Number of Parameters	Model Size	mAP	Length of Training
YOLOv8s + Fasternet	9,458,019	19.40	96.1	3.42
YOLOv8s + MobileNet	14,352,627	29.0	96.7	5.03
YOLOv8s + MobileNetv2	11,211,475	22.7	96.1	4.93
YOLOv8s + MobileNetv3	12,988,147	26.2	96.2	5.05
YOLOv8s + ResNet18	13,102,067	26.4	96.1	4.14
YOLOv8s + ResNet50	11,914,007	28.4	96.5	4.53
YOLOv8s + ShuffleNetv2	6,387,883	15.3	92.4	3.72
YOLOv8s + ConvNext	9,938,291	20.1	96.5	4.92

**Table 2:** Comparison of results of different feature fusion networks.

Network Model	Number of Parameters	Model Size	mAP	Length of Training
A + QAFPN	6,071,008	12.30	94.6	2.49
A + BiFPN	9,458,043	19.10	95.6	2.83
A + GhostsilmFPN	9,426,451	19.20	95.3	2.82
A + AsymptoticFPN	6,575,400	13.40	94.7	2.69
A + GiraffeDetFPN	9,356,787	18.90	95.5	2.81

**Table 3:** Results of ablation experiments. A is YOLOv8s, B is FasterNet, C is ECIoULoss, D is Rep-PAN, and E is QAFPN.

Exper Iment	A	B	C	D	E	Number of Parameters	GFLOPs	Length of Training	Model Size	mAP	Speed	Frame Rate
1	√					11,135,987	28.60	3.70	22.50	95.8	6.3	37.0
2	√	√				9,458,019	24.50	3.42	19.10	96.1	2.8	41.3
3	√		√			11,135,987	28.60	3.92	22.50	96.7	2.4	38.1
4	√			√	√	8,806,099	22.20	4.45	17.80	96.0	3.8	55.0
5	√	√	√			9,458,019	24.50	3.74	19.10	95.4	2.2	48.2
6	√	√	√	√		6,071,776	16.90	2.55	12.40	94.4	1.3	51.6
7	√	√	√	√	√	6,071,008	16.90	2.49	12.30	94.6	1.3	52.1

**Table 4:** Comparison results with lower version of YOLO model.

Network Model	Model Size/MB	Average Confidence Level/%	mAP/%	Frame Rate/fps
Ours	12.4	86.5	94.6	51.6
YOLOv8s	22.5	86.9	95.8	37.0
YOLOv7	72.1	86.1	94.9	35.93
YOLOv6	36.3	82.3	92.2	29.05

**Table 5:** Performance comparison of different deployment methods for models before and after improvement.

Deployment Method	YOLOv8s Frame Rate/fps	Ours Frame Rate/fps
Pytorch	2.4	3.8
Onnx	3.5	5.6
Ncnn	8.2	15.1
Tfilte	3.4	5.4

## Data Availability

The data presented in this study are available on request from the corresponding author.
